# Planar structures of medium-sized gold clusters become ground states upon ionization

**DOI:** 10.1039/d6na00102e

**Published:** 2026-05-22

**Authors:** Mohammad Ismaeil Safa, Ehsan Rahmatizad Khajehpasha, Jonas A. Finkler, Stefan Goedecker

**Affiliations:** a Department of Physics, University of Basel Klingelbergstrasse 82 CH-4056 Basel Switzerland stefan.goedecker@unibas.ch

## Abstract

This study investigates the structural stability of ionized gold clusters of sizes ranging from 22 to 100 atoms, contrasting compact, cage and planar structures. While it is well known that neutral clusters in the upper part of this size range predominantly favor compact structures, our results reveal that positively ionized gold clusters exhibit structural transitions in which planar structures become energetically preferred once the charge is sufficiently large. In addition, we study the finite-temperature stability of the structures and find that thermodynamic effects further stabilize planar structures relative to their compact counterparts. To explore the potential energy surface, we use the Minima Hopping algorithm combined with a machine-learned potential. Since the machine-learned potential does not apply to ionized clusters, we introduce a charge-correction term to incorporate Coulomb interactions and charge screening.

## Abstract 2

Ionization causes a transformation from compact to planar ground states in gold clusters.

## Graphical abstract

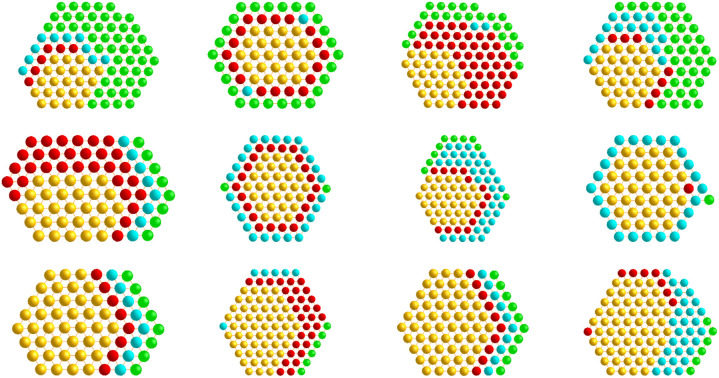

## 1 Introduction

Extensive experimental and theoretical studies show that small gold clusters exhibit a preference for planar (2D) structures up to a critical size, beyond which three-dimensional (3D) compact structures, (*i.e.* structures which can not host an extra endohedral atom), become energetically favorable.^1–4^ For anionic clusters, photoelectron spectroscopy, ion mobility measurements and Density Functional Theory (DFT) calculations consistently show that clusters remain planar up to Au_11_, with a structural transition occurring at Au_12_ where planar and 3D isomers coexist and clusters of Au_*n*≥13_ are 3D geometries.^1,3,5,6^ DFT predictions for the 2D to 3D crossover in neutral clusters are strongly method-dependent: Local Density Approximation (LDA) favors a transition at Au_7_, whereas Generalized Gradient Approximation (GGA) with scalar-relativistic can stabilize planar motifs up to Au_11_.^7^ Cage motifs emerge for Au_10_–Au_14_, followed by a transition to compact structures at Au_15_.^4^ Finite-temperature free-energy calculations using van der Waals-corrected DFT predict that Au_9,10_ remain planar at 100 K, whereas Au_11_ adopts a non-planar capped trigonal prism with *D*_3h_ symmetry; increasing temperature generally shifts populations toward non-planar structures for Au_8_–Au_13_, with Au_11_ being a notable exception due to many accessible near-degenerate planar isomers.^8^ A recent machine-learning-based global search has further refined this picture by systematically investigating Au_2_–Au_55_ clusters, identifying the planar to 3D transition at Au_14_ and a subsequent cage to core–shell transformation for Au_*n*≥26_.^9^ These results not only confirm earlier DFT-based trends but also provide a comprehensive, size-resolved structural map, including several previously unreported low-energy isomers for Au_13_ and Au_15_–Au_18_. Additionally, photoelectron spectroscopy and global search studies revealed that anionic clusters Au_16_^−^−Au_18_^−^ can also form experimentally detectable cages.^10^ A paradigmatic example is Au_20_, which adopts a tetrahedral structure, experimentally confirmed *via* photoelectron spectroscopy, shown to be a slightly distorted fragment of the bulk fcc lattice with remarkable stability due to its large HOMO–LUMO gap and high electron affinity.^11^ For Au_26_ and Au_26_^−^, global searches reveal a fluxional character. Many distinct minima are only ∼0.5 eV above the putative ground state and are separated by low barriers. In addition, the predicted ground state motif depends on the exchange–correlation (XC) functional: LDA favors filled-cage core–shell structures for Au_26_ and Au_26_^−^, whereas Perdew–Burke–Ernzerhof (PBE).^12^ Stabilizes an empty cage for Au_26_ and a tubular motif for.Au_26_^−^.^13^ In line with this picture, high-level coupled-cluster benchmarks for Au_27_^*q*^ already reveal an extremely shallow Potential Energy Surface (PES) with many quasi-degenerate compact and non-compact isomers and a strong XC functional dependence of the predicted ground state.^14^ For Au_32_, a cage with icosahedral symmetry satisfying the 2(*N* + 1)^2^ spherical aromaticity rule^15^ and exhibiting a large HOMO–LUMO gap is proposed as a “golden fullerene”.^16^ Stability, arising from aromaticity and a large HOMO–LUMO gap, was also found for Au_50_, where a DFT-based comparison of cage and compact structures found that a cage with *D*_6d_ was the global minimum.^17,18^ Au_42_ with icosahedral and Au_60_ with chiral icosahedral symmetry cages are typically slightly higher in energy than compacts, remain structurally robust local minima and can act as components in nested or multi-shell clusters.^19,20^ A study based on icosahedral-inspired templates showed that large quasi-icosahedral cages such as Au_92_ and Au_122_, although not global minima, are low-lying energy metastable structures with high symmetry and strong metallicity.^21^ Clusters in the Au_*n*∼100_ size range are compact, and entirely different structural motifs-such as icosahedral, decahedral, or octahedral-can be nearly degenerate in energy. As a consequence, the ground-state structural motif can change upon the addition of a single atom, and the ground-state structure oscillates among these motifs with increasing cluster size.^22^

While early studies primarily focused on small clusters and their transition from planar to compact geometries, recent experimental studies have demonstrated that atomically thin, free-standing gold monolayers can be synthesized using ligand-assisted self-assembly,^23^ intercalation beneath graphene,^24^ exfoliation from layered precursors^25^ and *in situ* dealloying inside electron microscopes.^26,27^ These monolayers exhibit remarkable thermal stability, metallic conductivity, and even magnetic edge states in nanoribbons. Moreover, studies based on DFT predicted that hexagonally close-packed 2D gold is dynamically^28^ and thermodynamically^29^ stable. Systematic DFT calculations indicate that graphene pores can stabilize free-standing 2D metal patches up to ∼8 nm^2^, and identify Au as one of the most promising elemental metals for forming stable pore-confined 2D phases.^30^ Complementary computational simulations using *ab initio* and molecular dynamics methods further reveal unique behaviors such as 2D liquid phases,^31^ strain-induced electronic transitions,^24^ and strong environment-dependent catalytic activity.^32–34^ These findings establish that planar gold structures are not restricted to small clusters but constitute a broader and experimentally realizable class of 2D materials.

These results collectively suggest that planar and cage motifs represent an important class of stable and potentially synthesizable configurations for medium-sized and large gold clusters. We therefore explored whether positive ionization, hereafter referred to simply as ionization, of electron-rich gold clusters provides a route to stabilizing planar or cage motifs. The rationale is based on the assumption that the positive charge is distributed over all atoms, leading to a repulsion between the ionic nuclei. Hence, the electrostatic energy will be smaller in a planar or cage structure, where the number of nearest neighbors is smaller than in compact structures. To test this hypothesis, we systematically investigated the PES of ionized gold clusters ranging from Au_22_ to Au_100_. For this purpose, we employed the Minima Hopping (MH) algorithm.^35^

Our approach uses the Atomic Simulation Environment (ASE)-integrated implementation of MH^36^ and symmetry-biased MH.^37^ We used machine-learned potentials to evaluate the energies and forces during the MH exploration, with a physically motivated charge-correction term introduced to account for coulombic repulsion and charge-screening effects in the ionized gold clusters considered here. Notably, our results reveal that planar geometries emerge as ground state configurations for certain ionization levels, suggesting that ionization can play a pivotal role in stabilizing non-compact cluster morphologies. Furthermore, we account for vibrational contributions to the free energy at finite temperatures. Since atomic vibrations are always present at non-zero temperatures, the vibrational free energy term can affect structural stability. Our calculations indicate that the vibrational free energy contribution at finite temperature further stabilizes planar geometries over compact counterparts. This suggests that, in addition to electrostatic effects, thermal fluctuations also promote the emergence of planar structures at room temperature.

## 2 Methods

### 2.1 Global structure search with MH and machine-learned potentials

The exploration of the PES for neutral and ionized gold clusters was carried out with the MH algorithm. MH alternates short molecular-dynamics escape moves and local geometry relaxations, while an adaptive temperature scheme controls the kinetic energy used in the escape steps, which makes it well-suited for sampling complex, high-dimensional PES and locating low-energy minima while avoiding excessive revisits of already known structures. In all MH simulations, the interatomic forces and energies were calculated by machine-learned interatomic potentials rather than by DFT, allowing us to perform extensive global searches for cluster sizes up to Au_100_.

As a primary PES model, we employed Neural Equivariant Interatomic Potential (NequIP),^38^ an *E*(3)-equivariant neural network potential that we trained on DFT reference data for gold clusters. With this potential, we could find all previously known neutral cage structures. By construction, this potential does not explicitly include electrostatic contributions associated with ionization. To extend its applicability to ionized clusters, we augmented NequIP with an electrostatic correction term that is added to both the total energy and the atomic forces.

We assumed that, upon ionization, the charge is uniformly distributed over all atomic sites, such that each nucleus carries an effective charge *Q*_tot_/*n*, where *Q*_tot_ is the total cluster charge and *n* is the cluster size. In our first attempt, we added a bare Coulomb interaction between these point charges on top of the NequIP outputs, but this unscreened correction overestimated the effect of ionization and did not reproduce the DFT trends for ionized clusters. We therefore introduced a screened interaction with an exponential damping factor to mimic charge screening and finite-size effects in the metallic cluster. The resulting energy correction term in atomic units takes the form:1
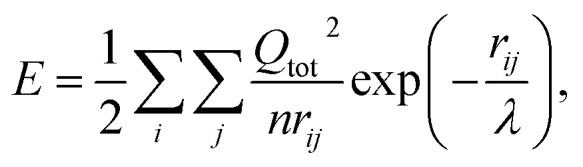
where *r*_*ij*_ is the distance between atoms *i* and *j*, and *λ* is an effective screening length that determines how rapidly the correction term decays with interatomic distance. Its value was obtained empirically by fitting to DFT reference energies for even-sized ionized Au clusters from Au_22_ to Au_100_ and ionization levels in the range of +2e to +10e in steps of 2*e*, resulting in an optimal global value of *λ* = 2.64 Å. The corresponding interatomic forces are then the negative gradient of this energy. The exponential damping takes into account screening effects, leading to a smoother and more physical description of nuclear repulsion inside the cluster. In all MH simulations, these correction terms were evaluated on-the-fly and added to the underlying NequIP energies and forces, so that the search is effectively performed on a charge-corrected PES.

Alongside the charge-corrected NequIP-based PES, we employed a pretrained MACE foundation model^39^ as a complementary machine-learned potential. Unlike NequIP, which was trained on our own dataset containing compact, cage, and planar structures and then augmented with the charge-correction term, the MACE model was not retrained on our dataset and did not include an explicit charge correction. We therefore did not use MACE on the same footing to describe ionized clusters. Instead, we performed separate MH simulations with MACE mainly to generate additional candidate structures, in particular compact motifs.

The low-energy structures obtained with NequIP and MACE were subsequently validated with DFT, providing evidence that the emergence of cage and planar ground states is not an artefact of machine-learned potentials.

### 2.2 DFT calculations

A large number of low-energy compact, cage and planar structures from MH outputs were selected to be validated by DFT. To accurately evaluate the energies and structural properties of ionized gold clusters, we performed DFT calculations using the FHI-aims package.^40^ FHI-aims employs an all-electron, numeric atom-centered Gaussian basis set, allowing for highly accurate electronic structure calculations. Relativistic effects are treated by the AZORA method.^41^

We employed three XC functionals to validate the robustness of the structural trends: PBE, its solid-state revision, PBEsol, combined with a many-body dispersion correction (PBEsol + MBD), both within the GGA framework; and r^2^SCAN within the *meta*-GGA framework.^12,42–44^

We employed the “tight” basis set to ensure high numerical accuracy in describing the electronic structure and treated all clusters using free boundary conditions, simulating isolated gas-phase systems without periodic constraints. Performing structural relaxations for neutral clusters and ionization levels up to +13e enabled us to analyze how increasing ionization drives structural transitions. We set the structural-optimization convergence threshold such that the force norm on each atom falls below 5 meV Å^−1^. These calculations provide reference data for benchmarking the machine-learned potentials and analyzing charge-dependent structural transitions in gold nanoclusters.

## 3 Results

In this study, we examined how the structures of gold clusters evolve with increasing ionization. As the ionization level increases, gold clusters show a tendency to form non-compact structures either in the form of a cage or of a planar structure. For selected Au_*n*_ clusters with *n* ≤ 100 atoms, we successively increased the ionization level until we could observe a transition of the ground state structure into a cage or planar structure. To characterize the stability of these ionization-induced ground states, we also analyzed the structural energy gap, defined here as the energy difference between the global minimum and the first metastable structure. A larger structural energy gap indicates a stronger energetic stabilization of the corresponding non-compact ground state. While a tendency to form planar structures upon ionization is always observed, different XC functionals predict different threshold ionizations for this transition. The results for the three widely used XC functionals we employed are presented below.

### 3.1 PBE

**Fig. 1:**
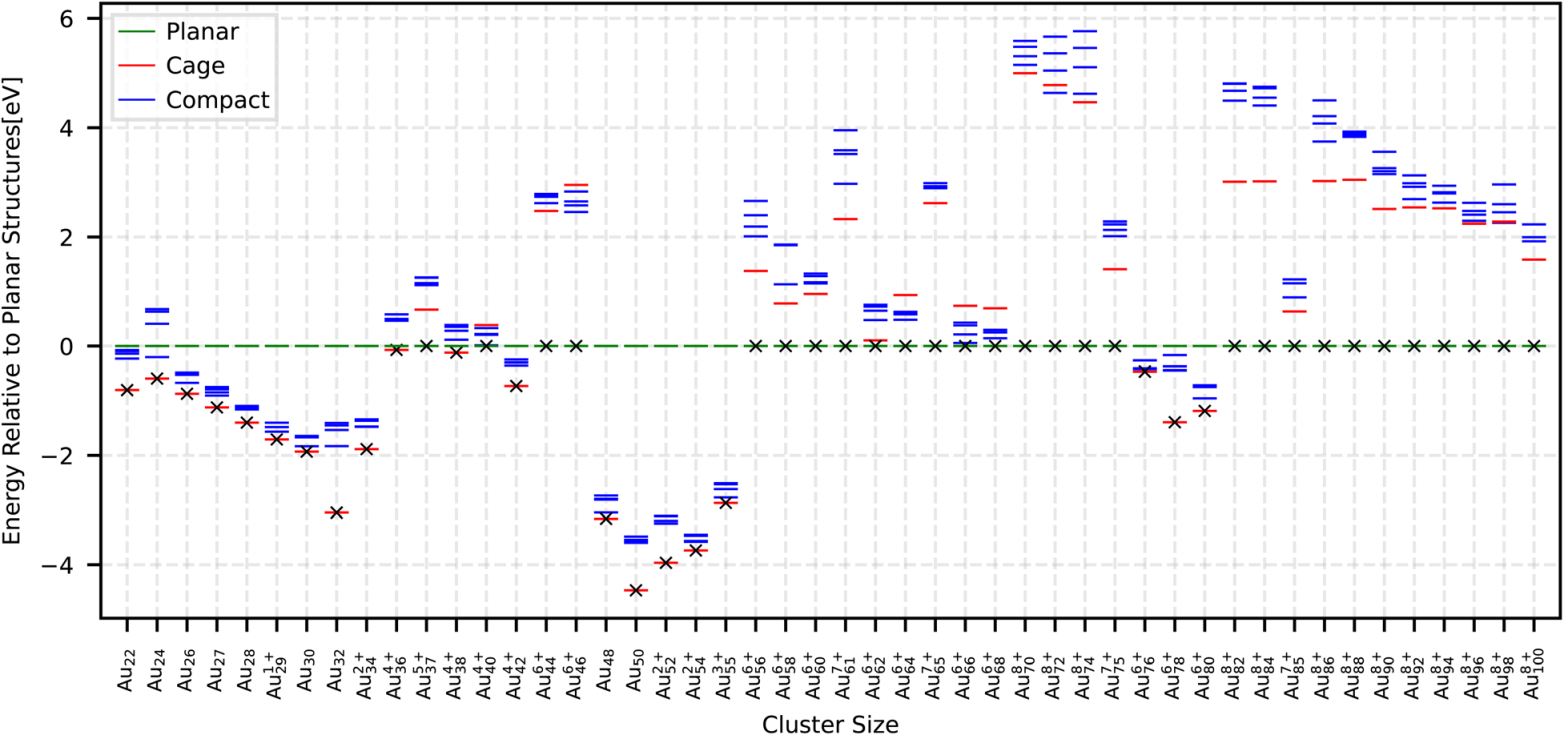
Relative energies with respect to the planar structure for the smallest ionization level at which a cage or planar structure becomes the ground state within the PBE XC functional. The global minimum is marked by a cross sign.

We employed PBE, a widely used non-empirical semilocal GGA XC functional. Among the three XC functionals considered in this work, PBE requires the lowest ionization level to stabilize non-compact motifs as ground states. In the neutral clusters, cage geometries are obtained as ground states for Au_*n*_ with *n* = 22, 24, 26, 27, 28, 30, 32, 48, and 50. Upon weak ionization, cages become the ground state for Au_29_^+1^, Au_34_^+2^, Au_36_^+4^, Au_38_^+4^, Au_42_^+4^, Au_52_^+4^, Au_54_^+2^ and Au_55_^+3^. At intermediate ionization level, cage ground states are found for Au_76_^+6^, Au_78_^+6^, and Au_80_^+6^. All remaining clusters favor planar structure as their ground state structures at the corresponding ionization levels. For the planar ground states, the structural energy gaps are as follows: Au_70_^+8^, Au_72_^+8^, Au_74_^+8^ fall between 4–5 eV; Au_82_^+8^, Au_84_^+8^, Au_86_^+8^ fall between 3–4 eV; Au_44_^+6^, Au_46_^+6^, Au_61_^+7^, Au_90_^+8^, Au_92_^+8^, Au_94_^+8^, Au_96_^+8^ and Au_98_^+8^ fall between 2–3 eV; Au_56_^+6^, Au_75_^+7^ and Au_100_^+8^ fall between 1–2 eV; all remaining cases fall between 0–1 eV. The detailed PBE relative energies and ground state assignments as a function of size and ionization level are shown in Fig. 1; the total energy of the lowest energy compact, cage and planar structures are shown in Table SII SI.

### 3.2 PBEsol + MBD

**Fig. 2:**
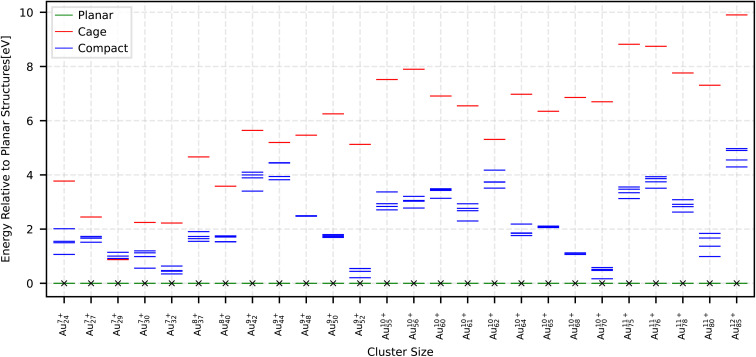
Relative energies with respect to the planar structure for the smallest ionization level at which the planar structure becomes the ground state within the PBEsol + MBD XC functional. The global minimum, which is a planar structure for all cases, is marked by a cross sign.

PBEsol is a semi-local GGA XC functional tailored for solids, and the MBD adds an explicit many-body dispersion correction. We performed separate relaxations of the same structural candidates to determine, for each size, the smallest ionization level at which a planar structure becomes the global minimum. The ionization level required for planar structures to become ground states is generally higher than that predicted by the other two XC functionals, while the corresponding structural energy gaps are as follows: Au_85_^+12^ with 4.29 eV has the largest structural energy gap; Au_42_^+9^, Au_44_^+9^, Au_60_^+10^, Au_62_^+10^, Au_75_^+11^ and Au_76_^+11^ fall between 3–4 eV; Au_48_^+9^, Au_55_^10^, Au_56_^+10^, Au_61_^+10^, Au_65_^+10^ and Au_78_^+11^ fall between 2–3 eV; Au_24_^+7^, Au_27_^+7^, Au_37_^+8^, Au_40_^+8^, Au_50_^+9^, Au_64_^+10^ and Au_68_^+10^ fall between 1–2 eV; all remaining cases fall between 0–1 eV. This XC functional does not favor cage motifs at all; whenever the planar structures become ground state, the cages are higher in energy than compacts. In the Table 1, the ionization levels required for each cluster size are reported. The resulting energetic ordering is shown in Fig. 2. The total energy of the lowest energy compact, cage and planar structures are shown in the Table SIII SI.

**Table 1:** For each cluster size, the table reports for the r^2^SCAN, PBEsol + MBD, and PBE XC functionals the ionization level *Q* at which the lowest-energy non-compact motif first becomes energetically competitive with the compact minimum, together with the point-group symmetry of the corresponding lowest-energy compact and cage structures at that *Q*

Cluster	PBE			PBEsol + MBD			r^2^SCAN		
	*Q*	Compact	Cage	*Q*	Compact	Cage	*Q*	Compact	Cage
Au_24_	0	*C*_s_	*C*_2v_	8	*C*_2v_	*C*_2v_	0	*C*_1_	*C*_2v_
Au_27_	0	*C*_2v_	*D*_3h_	7	*C*_s_	*D*_3h_	0	*C*_s_	*C*_s_
Au_29_	1	*C*_3v_	*D*_3h_	7	*C*_s_	*D*_3h_	1	*C*_1_	*D*_3h_
Au_30_	0	*C*_1_	*C*_1_	7	*C*_s_	*C*_1_	5	*C*_1_	*C*_1_
Au_32_	0	*C*_1_	*I*_*h*_	7	*C*_3_	*C*_1_	0	*C*_s_	*I*_h_
Au_37_	5	*C*_1_	*D*_5h_	8	*C*_1_	*C*_2_	5	*C*_s_	*D*_5h_
Au_40_	4	*C*_1_	*C*_1_	8	*C*_1_	*C*_1_	6	*C*_1_	*C*_s_
Au_42_	4	*C*_1_	*D*_5d_	9	*C*_1_	*C*_1_	6	*C*_1_	*D*_5d_
Au_44_	6	*C*_s_	*C*_1_	9	*C*_1_	*C*_1_	6	*C*_s_	*C*_1_
Au_48_	0	*C*_s_	*C*_2_	9	*C*_s_	*C*_1_	6	*C*_s_	*C*_2_
Au_50_	0	*C*_1_	*D*_6d_	9	*C*_1_	*D*_6d_	7	*C*_3_	*D*_6d_
Au_52_	2	*C*_1_	*C*_1_	9	*C*_2v_	*C*_1_	6	*C*_1_	*C*_1_
Au_55_	3	*C*_1_	*C*_1_	10	*C*_s_	*C*_1_	6	*C*_1_	*C*_1_
Au_56_	6	*C*_1_	*C*_1_	10	*C*_1_	*C*_1_	7	*C*_1_	*C*_1_
Au_60_	6	*C*_2_	*C*_2_	10	*C*_1_	*C*_2_	7	*C*_1_	*C*_2_
Au_61_	7	*C*_3_	*C*_1_	10	*C*_1_	*C*_1_	8	*C*_1_	*C*_1_
Au_62_	6	*C*_3_	*D*_5d_	10	*C*_3_	*D*_5d_	8	*C*_3_	*D*_5d_
Au_64_	6	*C*_2_	*C*_1_	10	*C*_1_	*C*_1_	8	*C*_1_	*C*_1_
Au_65_	7	*C*_1_	*C*_1_	10	*C*_1_	*C*_1_	8	*C*_1_	*C*_1_
Au_68_	6	*C*_1_	*C*_1_	10	*C*_2_	*C*_1_	8	*D*_2_	*C*_1_
Au_70_	8	*C*_1_	*C*_1_	10	*C*_1_	*C*_1_	8	*C*_1_	*C*_1_
Au_75_	7	*C*_2_	*C*_2_	11	*C*_1_	*D*_2_	8	*C*_2_	*C*_2_
Au_76_	6	*C*_1_	*C*_1_	11	*C*_1_	*C*_1_	8	*C*_1_	*C*_1_
Au_78_	6	*C*_1_	*T*_h_	11	*C*_1_	*T*_h_	8	*C*_1_	*T*_h_
Au_80_	6	*C*_1_	*C*_1_	11	*C*_1_	*C*_2_	8	*C*_1_	*C*_2_
Au_85_	7	*C*_1_	*C*_1_	12	*C*_1_	*C*_1_	9	*C*_1_	*C*_1_

### 3.3 r^2^SCAN

**Fig. 3:**
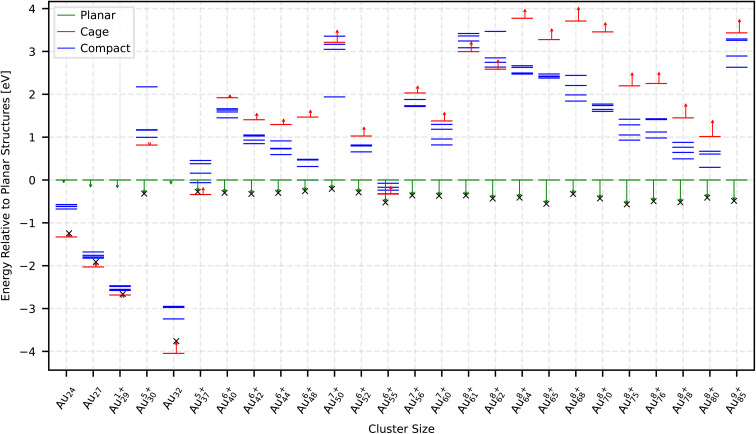
Relative energies with respect to the planar structure for the smallest ionization level at which a cage or planar structure becomes the ground state within the r^2^SCAN XC functional. The plot also includes vibrational free energy contribution at 300 K, shown as arrows whose amplitudes represent the vibrational free energy differences relative to the vibrational free energy of the lowest-energy compact structure. A downward arrow indicates that inclusion of the vibrational free energy term stabilizes the corresponding structure, while an upward arrow indicates the opposite. The global minimum marked by a cross sign at the tip of the arrows.

r^2^SCAN is a non-empirical, numerically regularized *meta*-GGA in the SCAN family that improves numerical stability while satisfying the most important known exact constraints for *meta*-GGA XC functionals. It builds on earlier regularization efforts rSCAN^45^ that were introduced to mitigate SCAN's^46^ numerical sensitivity, while SCAN itself was originally designed as a broadly constrained *meta*-GGA. Within the r^2^SCAN description, cage geometries are obtained as ground states for the neutral clusters Au_24_, Au_27_, and Au_32_. Upon ionization, cages become the ground state for Au_29_^+1^, Au_37_^+5^, and Au_55_^+6^, whereas for all other investigated clusters, planar structures are stabilized as ground states at the corresponding ionization levels. Overall, the ionization level required to favor planar structures in r^2^SCAN lies between those predicted by PBE and by PBEsol + MBD, *i.e.*, higher than in PBE but lower than in PBEsol + MBD. For planar ground states, the structural energy gaps are as follows: Au_61_^+8^, Au_62_^+8^, Au_64_^+8^, Au_65_^+8^ and Au_85_^+9^ fall between 2–3 eV; Au_40_^+6^, Au_50_^+7^, Au_56_^+7^, Au_68_^+8^ and Au_70_^+8^ fall between 1–2 eV; all remaining cases fall between 0–1 eV. In the Table 1, the ionization levels required to stabilize non-compact motifs as ground states are listed. The resulting energetic ordering is shown in Fig. 3. The total energy of the lowest energy compact, cage and planar structures are shown in the Table SIV SI.

### 3.4 Density of states

The compact structures have a very high density of states, *i.e.*, there exist many nearly degenerate structures. For each ionization level, we showed only the four lowest-energy compact structures. In general, there is a substantial structural energy gap between the planar global minimum and the lowest compact cluster as seen in Fig. 1–3. So even if there was a slightly lower compact structure, it is very unlikely that it would be lower than the planar structures.

### 3.5 Temperature effects

We investigated the effects of temperature on the stability of planar structures by calculating the vibrational free energy at finite temperature. The results show that temperature effects further stabilize the planar geometries relative to their compact and cage counterparts. Fig. 3 shows the free energies with respect to planar geometries. It can be seen that taking into account the temperature effects changes the global minimum of Au_37_ and Au_55_ from a cage to a planar configuration. In addition, the structural energy gap between planar geometries and the first metastable geometries get larger. The vibrational free energies for Au_50_ to Au_85_ are calculated by charge-corrected NequIP and for the rest by DFT using the r^2^SCAN XC functional at 300 k.

### 3.6 Building patterns

**Fig. 4:**
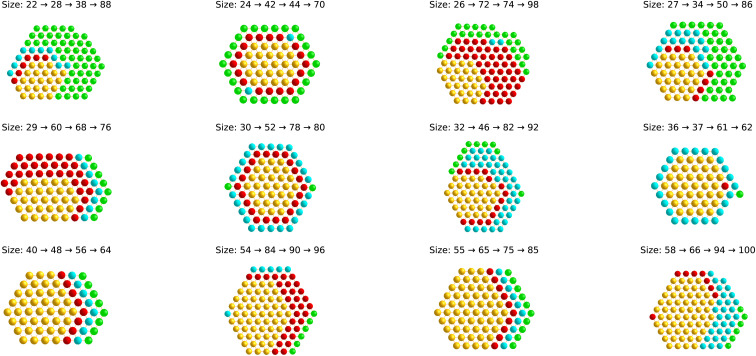
Planar configurations of minimally ionised gold clusters of all investigated sizes. The atoms are always arranged in a regular, close-packed hexagonal pattern. Progression from one size to the next is obtained by successively adding the atoms highlighted in red, then blue, and finally green, to the atoms shown in gold.

To better understand the planar structural patterns, we analyzed their building patterns across multiple cluster sizes. The growth is not always achieved by completing full shells; in many cases, atoms are added selectively along edges or specific directions, leading to anisotropic yet regular structures as shown in Fig. 4. For example, the transition from 55 to 85 atoms involves additions that extend particular edges rather than forming uniform shells. Even though the required minimum ionization level for obtaining planar structures varies for different XC functionals, the lowest energy planar structures of all XC functionals have the same overall structure. The different ionization levels and XC functionals just lead to barely visible structural relaxations.

### 3.7 Symmetries

**Fig. 5:**
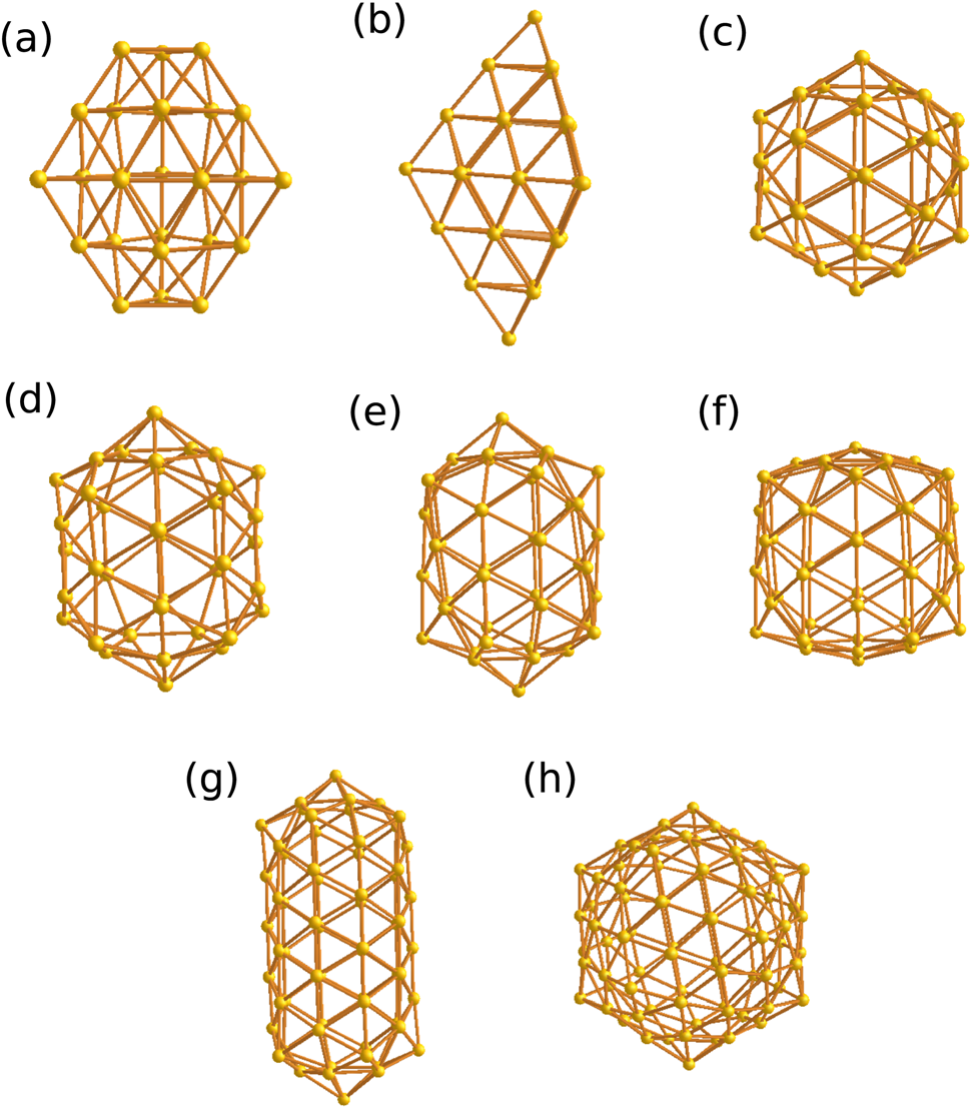
Subfigure (a)–(h) show the highly symmetric cage structures for Au_27_, Au_29_, Au_32_, Au_37_, Au_42_, Au_50_, Au_62_ and Au_78_ respectively.

In Fig. 5 we highlight high-symmetry cage structures, Au_32_ (*I*_h_), Au_27,29_ (*D*_3h_), Au_37_ (*D*_5h_), Au_42,62_ (*D*_5d_), Au_50_ (*D*_6d_) and Au_78_ (*T*_h_). The assigned point-group symmetries, however, are XC functional dependent. For PBE and r^2^SCAN, these cages largely retain their high symmetry character; the only notable exception is Au_27_, which relaxes from *D*_3h_ (PBE) to *C*_s_ (r^2^SCAN). With PBEsol + MBD, Au_32,37,42_ clusters relax into strongly distorted cages and lose their symmetries. Intermediate symmetries are observed in: Au_22,24,26_ (*C*_2v_), Au_28_ (*C*_3v_) and Au_34,75_ (*D*_2_). The rest of the cages, however, adopt low symmetry and are classified as *C*_s_, *C*_2_ and *C*_1_. Table 1 shows for the clusters that we calculated them with three XC functionals (with PBE XC functional we have calculated more clusters than the other two), the ionization level at which non-compact structures become energetically competitive, together with the point-group symmetry of the corresponding compact and cage at that ionization level for r^2^SCAN, PBEsol + MBD and PBE XC functionals. Taken together, these results demonstrate that while cage motifs frequently adopt highly symmetric forms, the compact structures favor distorted, low-symmetry configurations. Being trained on bulk materials, MACE gave only compact structures, but no low-energy cages. Finally, we note that the point-group symmetries reported here arise from the structures relaxed with a standard finite electronic smearing (Gaussian broadening) of 0.01 eV. In the zero-electronic-temperature limit, degenerate states at the Fermi level drive small Jahn–Teller distortions that lift the degeneracy by reducing the symmetry, and slightly lower the total energy. The distortions are, however, so small that they can hardly be seen by eye. In the resulting structures, all electronic levels are completely filled with integer occupation numbers, so the rule that determines the structure of small clusters, namely that clusters adopt shapes such that electronic shells can be filled completely,^47^ is also applicable to larger clusters.

## 4 Conclusions

In this study, we showed that ionization can transform the ground states of medium-sized clusters from compact bulk-like structures to planar structures consisting of hexagonal honeycomb patterns. These planar structures are considerably lower in energy than ionized compact structures of the same size. Weaker ionizations can also lead to cage structures. The predicted degree of ionization that is necessary to induce cage or planar ground states, unfortunately, depends on the XC functional that is used. We found that the PBE XC functional requires the smallest amount of ionization to induce planar ground states. The r^2^SCAN requires a somewhat larger ionization and PBEsol + MBD requires the largest amount. The r^2^SCAN XC functional, which also includes dispersion interactions, would probably be considered by most researchers as the most accurate XC functional. It yields results in closest agreement with experiment. PBEsol + MBD predicts compact ground states for all neutral clusters. For some sizes such as Au_24_, Au_27_ and Au_32_ there are however experimental indications that the ground states are cages.^14,16,48^ This is also predicted by the PBE and r^2^SCAN XC functionals. In contrast to experimental evidence, cages are never lower in energy than planar structures with the PBEsol + MBD XC functional. So we also consider in this context the r^2^SCAN results as the most reliable results. The ionization level given in Table 1 should therefore best match an experimental verification of our results. Although the minimum ionization level at which planar or cage structures become the global minima depends on the XC functional, the overall conclusion remains unchanged: ionization stabilizes non-compact structures across all functionals considered.

## Conflicts of interest

There are no conflicts to declare.

## Supplementary Material

NA-008-D6NA00102E-s001

NA-008-D6NA00102E-s002

## Data Availability

The coordinate files for all structures reported in this paper are deposited and are provided in the accompanying zipped archive. Further information regarding the data can be obtained by contacting the corresponding author *via* email. Supplementary information (SI) is available. See DOI: https://doi.org/10.1039/d6na00102e.
