# Supplementary material for: Planar structures of medium-sized gold clusters become ground states upon ionization
Source: Nanoscale Adv. 2026 May 22;8(12):3605–12. doi: 10.1039/d6na00102e (PMC13216818; doi:10.1039/d6na00102e)
Supplement: NA-008-D6NA00102E-s002 [file NA-008-D6NA00102E-s002.pdf]

Supplementary Information (SI) for Nanoscale Advances.
This journal is © The Royal Society of Chemistry 2026

Supplementary Information

0.1 Dataset and model setup

The training dataset contains a diverse set of gold clusters ranging from 10 to 140 atoms, including compact, planar, and cage structures. The DFT reference energies were computed with the Projector-Augmented Wave (PAW) method [1, 2] using 11 valence electrons as implemented in the Vienna Ab initio Simulation Package (VASP) [3, 4] together with the PBE exchange-correlation functional [5]. To remove interactions between periodic images, a vacuum of 12 Å was introduced in all directions around the nanoclusters. Figures S1 and S2 show the distribution of cluster sizes and total energies in the training set, respectively.

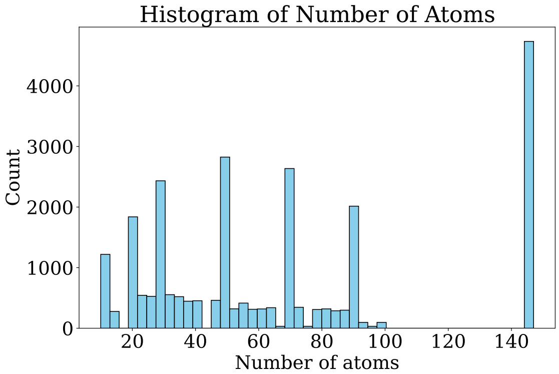

Fig. S1: Histogram of the number of atoms in the training dataset.

The NequIP model was trained for 82 epochs using a force-matching loss that combines force and energy contributions. The network architecture, radial cutoff, number of interaction blocks, and angular-momentum channels were chosen to balance accuracy and computational efficiency.

0.2 Performance and application

The final NequIP model reproduces the DFT reference data with high accuracy for both energies and forces and is therefore suitable as a surrogate potential for global structure searches. Detailed performance metrics, including root-mean-square errors (RMSEs) for energies and forces on the training and validation sets, are summarized in table SI. A comparison with other interaction models is shown in fig. S3.

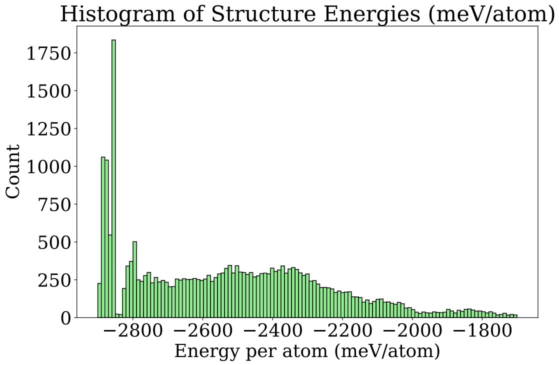

Fig. S2: Energy distribution of structures in the training dataset (meV/atom).

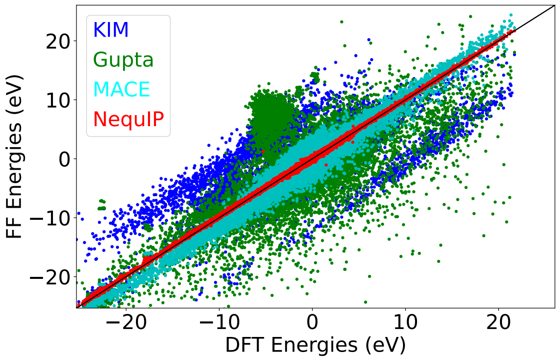

Fig. S3: Correlation of total energies by EAM method [6, 7], Gupta [8], MACE and NequIP models with DFT reference energies for a representative set of gold cluster configurations.

0.3 Validation of the charge-corrected NequIP potential

To assess whether the electrostatic correction preserves the quality of the underlying NequIP potential, we first compared DFT and charge-corrected

Table SI: Energy and force root-mean-square errors of the trained NequIP model on the training and validation sets.

	E_{RMSE} (eV)	F_{RMSE} (eV/Å)
Training	0.00541	0.0463
Validation	0.00441	0.0473

NequIP energies as a function of ionization level for representative clusters. Figures S4 and S5 show the energy difference per atom relative to the compact structures for Au_{76} and Au_{54}, respectively, over the charge range q = 0-10e. In both cases, the charge-corrected NequIP curves closely track the DFT trends: the energies decrease monotonically with increasing charge. The deviations between DFT and the corrected NequIP energies remain small compared to the total stabilization with charge, indicating that the added electrostatic term does not distort the global charge dependence of the PES.

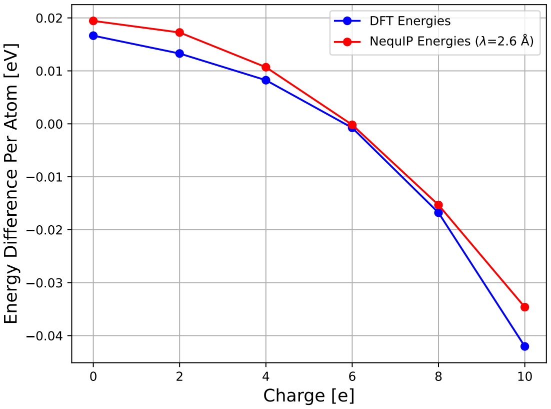

Fig. S4: Energy difference per atom as a function of charge for Au_{76}, comparing DFT and the charge-corrected NequIP potential. Energies are referenced to the compact structure.

Since the vibrational free energy depends on the curvature of the PES around the minimum, a comparison of vibrational free-energy differences provides an implicit but stringent test of the forces and their derivatives. Figures S6 and S7 show the vibrational free-energy differences at $T = 300$ K (including zero-point energy) between planar and compact structures, and between cage and compact structures, respectively. For all considered cluster sizes, the charge-corrected NequIP results closely follow the DFT values: the sign of the free-energy difference is identical in every case, the magnitude is reproduced to good accuracy, and the relative ordering of clusters is preserved. In particular, both DFT and charge-corrected NequIP predict a substantial lowering of the free energy for planar structures relative to compact ones, and a corresponding increase for cage structures relative to their compact counterparts.

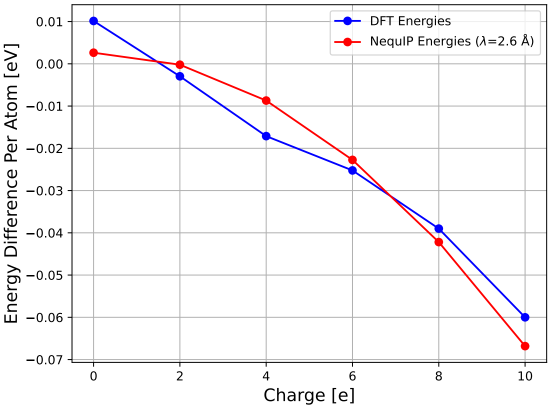

Fig. S5: Energy difference per atom as a function of charge for Au_{54}, comparing DFT and the charge-corrected NequIP potential. Energies are referenced to the compact structure.

Taken together, these comparisons demonstrate that the electrostatic correction preserves both the global charge dependence of the energy and the local curvature around relevant minima. The charge-corrected NequIP potential therefore provides a reliable description of energies and forces for the ionized gold clusters studied here.

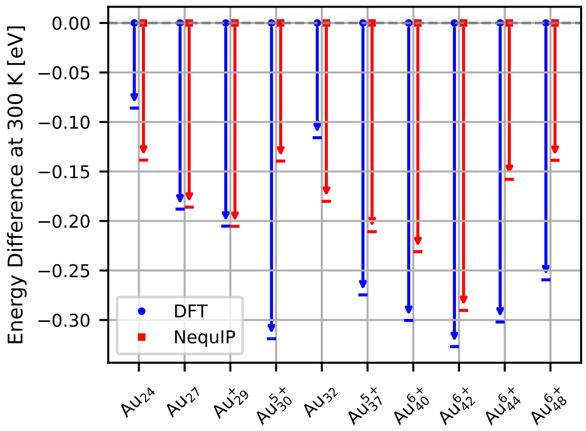

Fig. S6: Vibrational free-energy differences $\Delta F_{\mathrm{vib}}(300\ \mathrm{K})$ between planar and compact structures for selected clusters, comparing DFT and the charge-corrected NequIP potential. Negative values indicate that the planar structure is favoured at 300K.

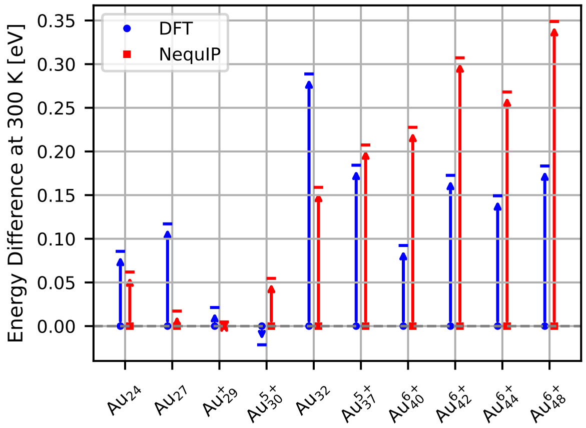

Fig. S7: Vibrational free-energy differences $\Delta F_{\mathrm{vib}}(300\ \mathrm{K})$ between cage and compact structures for selected clusters, comparing DFT and the charge-corrected NequIP potential. Positive values indicate that the cage structure is higher in free energy than the corresponding compact minimum at 300K.

Table SII: Total energies of the lowest-energy compact, cage and planar structures for the studied gold clusters at the indicated ionization level calculated with PBE XC functional in eV units.

Size	Q	E_{compact}	E_{cage}	E_{planar}
Au22	0	-11784373.8829949312	-11784374.4533069003	-11784373.6514568999
Au24	0	-12855681.3926772289	-12855681.7798710000	-12855681.1892409008
Au26	0	-13926988.7358446382	-13926988.9359470997	-13926988.0666028000
Au27	0	-14462642.3687242344	-14462642.5863713007	-14462641.4669601992
Au28	0	-14998296.1054223254	-14998296.3471069001	-14998294.9480936006
Au29	1	-15533943.9258133303	-15533944.0681103002	-15533942.3612922002
Au30	0	-16069603.8613478243	-16069603.9608851001	-16069602.0293128993
Au32	0	-17140911.2473042682	-17140912.4660142921	-17140909.4174401015
Au34	2	-18212203.5167145170	-18212203.9256322011	-18212202.0406000018
Au36	4	-19283485.7045718022	-19283486.2409333996	-19283486.1698722988
Au37	5	-19819123.7293879837	-19819124.1781540997	-19819124.8460233994
Au38	4	-20354793.7920847908	-20354794.0276058018	-20354793.9067832008
Au40	4	-21426101.5680953264	-21426101.1955628991	-21426101.5802452005
Au42	4	-22497409.3015969880	-22497409.6695428044	-22497408.9425473996
Au44	6	-23568683.3399797492	-23568683.4833232984	-23568685.9555942006
Au46	6	-24639991.4168872386	-24639990.9199844003	-24639993.8701508008
Au48	0	-25711371.6179627664	-25711371.7310128994	-25711368.5723331012
Au50	0	-26782679.0864743069	-26782679.9584648982	-26782675.4886697009
Au52	2	-27853972.1115852855	-27853972.8297604993	-27853968.8647666983
Au54	2	-28925279.8003975078	-28925279.9600207992	-28925276.2220741995
Au55	3	-29460923.1164041013	-29460923.2176709995	-29460920.3459875993
Au56	6	-29996531.6094097457	-29996532.2437016000	-29996533.6215785000
Au58	6	-31067840.1627144553	-31067840.5137103982	-31067841.2947059013
Au60	6	-32139148.0416897386	-32139148.2349390984	-32139149.1917935014
Au61	7	-32674783.2398122884	-32674783.8868740015	-32674786.2096567005
Au62	6	-33210456.3095684536	-33210456.6824061610	-33210456.7862414010
Au64	6	-34281764.5139424354	-34281764.0594263002	-34281764.9952920005
Au65	7	-34817399.5628386959	-34817399.8377309963	-34817402.4527482986
Au66	6	-35353072.6804527864	-35353071.9976650029	-35353072.7357299030
Au68	6	-36424380.4528341815	-36424379.9037233964	-36424380.5952892005
Au70	8	-37495649.5527808889	-37495649.7048689000	-37495654.6976476000
Au72	8	-38566958.2508317083	-38566958.1069963000	-38566962.8893812000
Au74	8	-39638266.3376686349	-39638266.4942546000	-39638270.9605859000
Au75	7	-40173939.9928744510	-40173940.5992190018	-40173942.0098382011
Au76	6	-40709612.0961799324	-40709612.1421656013	-40709611.6766050011
Au78	6	-41780919.9421525151	-41780920.8910811022	-41780919.4993818998
Au80	6	-42852228.1418293491	-42852228.3718110025	-42852227.1877057031
Au82	8	-43923498.3359515369	-43923499.8252495974	-43923502.8322952986
Au84	8	-44994806.2333708853	-44994807.6258789971	-44994810.6411446035
Au85	7	-45530480.3363316879	-45530480.5950400010	-45530481.2286486998
Au86	8	-46066114.7963887751	-46066115.5232174993	-46066118.5422165990
Au88	8	-47137422.8190271556	-47137423.6060635000	-47137426.6503728032
Au90	8	-48208731.0783924833	-48208731.7197723985	-48208734.2271434963
Au92	8	-49280039.5807077140	-49280039.7334377989	-49280042.2704674974
Au94	8	-50351347.5194993913	-50351347.6239837036	-50351350.1432097033
Au96	8	-51422655.7879557535	-51422655.8367601037	-51422658.0803427994
Au98	8	-52493963.7280049473	-52493963.7120855972	-52493965.9882744998
Au100	8	-53565271.7499916703	-53565272.0851155967	-53565273.6715193987

Table SIII: Total energies of the lowest-energy compact, cage and planar structures for the studied gold clusters at the indicated ionization level calculated with PBEsol+MBD XC functional in eV units.

Size	Q	E_{compact}	E_{cage}	E_{planar}
Au24	7	-12851992.8814177681	-12851990.1754461676	-12851993.9469800312
Au27	7	-14458508.9354776330	-14458508.0049921852	-14458510.4501365535
Au29	7	-15529520.1368721183	-15529520.1599955764	-15529521.0327065252
Au30	7	-16065025.6610906329	-16065023.9735914040	-16065026.2178222984
Au32	7	-17136036.1777288318	-17136034.2995584235	-17136036.5220437683
Au37	8	-19813538.1705333702	-19813535.0545214266	-19813539.7178942002
Au40	8	-21420053.9577834904	-21420051.9037468024	-21420055.4860454276
Au42	9	-22491039.1179469712	-22491036.8775570393	-22491042.5188986212
Au44	9	-23562049.4228905290	-23562048.0477586761	-23562053.2435088828
Au48	9	-25704071.2119783461	-25704068.2209315747	-25704073.6852728166
Au50	9	-26775082.5192736201	-26775077.9584287070	-26775084.2105447613
Au52	9	-27846094.1124891043	-27846089.1916963086	-27846094.3166619316
Au55	10	-29452584.0350670293	-29452579.2255310230	-29452586.7437124103
Au56	10	-29988089.1973293573	-29988084.0762551837	-29988091.9731848203
Au60	10	-32130109.3035199046	-32130105.5282131210	-32130112.4390639514
Au61	10	-32665614.8136003278	-32665610.5654794015	-32665617.1123959087
Au62	10	-33201118.7035499662	-33201116.9057762623	-33201122.2153994143
Au64	10	-34272131.2917612493	-34272126.0756461248	-34272133.0516912043
Au65	10	-34807635.6655190289	-34807631.3757642657	-34807637.7224883810
Au68	10	-36414152.3406183347	-36414146.5469158441	-36414153.4039350450
Au70	10	-37485163.1232914180	-37485156.5913205147	-37485163.2896568999
Au75	11	-40162662.4295440763	-40162656.7347052917	-40162665.5549834371
Au76	11	-40698167.5411698073	-40698162.3049923852	-40698171.0495504960
Au78	11	-41769177.6778317764	-41769172.5451158136	-41769180.3056200817
Au80	11	-42840189.5489087030	-42840183.2280374393	-42840190.5364795625
Au85	12	-45517688.6020401269	-45517682.9921225086	-45517692.8950748593

Table SIV: Total energies of the lowest-energy compact, cage and planar structures for the studied gold clusters at the indicated ionization level calculated with r^{2}SCAN functional in eV units.

Size	Q	E_{compact}	E_{cage}	E_{planar}
Au24	0	-12855515.9595729932	-12855516.6123223491	-12855515.2829093002
Au27	0	-14462456.7595318630	-14462456.9645614643	-14462454.9352743775
Au29	1	-15533744.6757797468	-15533744.7820909619	-15533742.0970994346
Au30	5	-16069338.7766732369	-16069338.9568433017	-16069339.7718810383
Au32	0	-17140691.7607096955	-17140692.5649791211	-17140688.5184396170
Au37	5	-19818869.7899332419	-19818870.0673865154	-19818869.7279463746
Au40	6	-21425792.6897237673	-21425792.2200301364	-21425794.1400935724
Au42	6	-22497087.4161620438	-22497086.8574161083	-22497088.2642474622
Au44	6	-23568382.1040482968	-23568381.4029078223	-23568382.6969524436
Au48	6	-25710971.3836712800	-25710969.9138409160	-25710971.3817078769
Au50	7	-26782245.8384333365	-26782244.5643433854	-26782247.7779425494
Au52	6	-27853559.4697657786	-27853559.1002398059	-27853560.1268005781
Au55	6	-29460501.4421241246	-29460501.4501994476	-29460501.1217344627
Au56	7	-29996129.1721618697	-29996128.8554048650	-29996130.8869261593
Au60	7	-32138718.5432152487	-32138717.9837394021	-32138719.3619752303
Au61	8	-32674344.6072562374	-32674344.6977852881	-32674347.6937589720
Au62	8	-33209992.2565247416	-33209992.3066731915	-33209994.8916107081
Au64	8	-34281287.5649208203	-34281286.2623199895	-34281290.0366618708
Au65	8	-34816934.5829489604	-34816933.6845940202	-34816936.9615447745
Au68	8	-36423877.1656726077	-36423875.2970955744	-36423879.0072333813
Au70	8	-37495171.4333114251	-37495169.5787350461	-37495173.0350215882
Au75	8	-40173407.8779937848	-40173406.6102867797	-40173408.8073367774
Au76	8	-40709055.1760824099	-40709053.9038519040	-40709056.1566343531
Au78	8	-41780349.7217550129	-41780348.7655952126	-41780350.2157986462
Au80	8	-42851644.5419136807	-42851643.8219444156	-42851644.8369588703
Au85	9	-45529859.4852932543	-45529858.6832017750	-45529862.1163276657

References

1 Peter E. Blöchl. Projector augmented-wave method. *Phys. Rev. B*, 50:17953–17979, 1994. doi: 10.1103/PhysRevB.50.17953.

2 Georg Kresse and Daniel Joubert. From ultrasoft pseudopotentials to the projector augmented-wave method. *Phys. Rev. B*, 59:1758–1775, 1999. doi: 10.1103/PhysRevB.59.1758.

3 Georg Kresse and Jürgen Furthmüller. Efficient iterative schemes for ab initio total-energy calculations using a plane-wave basis set. *Phys. Rev. B*, 54:11169–11186, 1996. doi: 10.1103/PhysRevB.54.11169.

4 Georg Kresse and Jürgen Furthmüller. Efficiency of ab-initio total energy calculations for metals and semiconductors using a plane-wave basis set. *Comput. Mater. Sci.*, 6:15–50, 1996. doi: 10.1016/0927-0256(96)00008-0.

5 John P. Perdew, Kieron Burke, and Matthias Ernzerhof. Generalized gradient approximation made simple. *Phys. Rev. Lett.*, 77:3865–3868, 1996. doi: 10.1103/PhysRevLett.77.3865.

6 Murray S. Daw and M. I. Baskes. Embedded-atom method: Derivation and application to impurities, surfaces, and other defects in metals. *Phys. Rev. B*, 29:6443–6453, 1984. doi: 10.1103/PhysRevB.29.6443.

7 C. J. O'Brien, C. M. Barr, P. M. Price, K. Hattar, and S. M. Foiles. Grain boundary phase transformations in ptau and relevance to thermal stabilization of bulk nanocrystalline metals. *J. Mater. Sci.*, 53:2911–2927, 2018. doi: 10.1007/s10853-017-1706-1.

8 Raju P. Gupta. Lattice relaxation at a metal surface. *Phys. Rev. B*, 23:6265–6270, 1981. doi: 10.1103/PhysRevB.23.6265.
